# Generation of Knock-In Pigs Carrying Oct4-tdTomato Reporter through CRISPR/Cas9-Mediated Genome Engineering

**DOI:** 10.1371/journal.pone.0146562

**Published:** 2016-01-12

**Authors:** Sisi Lai, Shu Wei, Bentian Zhao, Zhen Ouyang, Quanjun Zhang, Nana Fan, Zhaoming Liu, Yu Zhao, Quanmei Yan, Xiaoqing Zhou, Li Li, Jige Xin, Yangzhi Zeng, Liangxue Lai, Qingjian Zou

**Affiliations:** 1 CAS Key Laboratory of Regenerative Biology, South China Institute for Stem Cell Biology and Regenerative Medicine, Guangzhou Institutes of Biomedicine and Health, Chinese Academy of Sciences, Guangzhou, China; 2 Jilin Provincial Key Laboratory of Animal Embryo Engineering, Institute of Zoonosis, College of Veterinary Medicine, Jilin University, Changchun, China; 3 Key Laboratory of Banna Mini-pig Inbred Line of Yunnan Province, Animal Science and Technology College, Yunnan Agricultural University, Kunming, China; Beatson Institute for Cancer Research Glasgow, UNITED KINGDOM

## Abstract

The porcine pluripotent cells that can generate germline chimeras have not been developed. The Oct4 promoter-based fluorescent reporter system, which can be used to monitor pluripotency, is an important tool to generate authentic porcine pluripotent cells. In this study, we established a porcine Oct4 reporter system, wherein the endogenous Oct4 promoter directly controls red fluorescent protein (RFP). 2A-tdTomato sequence was inserted to replace the stop codon of the porcine Oct4 gene by homogenous recombination (HR). Thus, the fluorescence can accurately show the activation of endogenous Oct4. Porcine fetal fibroblast (PFF) lines with knock-in (KI) of the tdTomato gene in the downstream of endogenous Oct4 promoter were achieved using the CRISPR/CAS9 system. Transgenic PFFs were used as donor cells for somatic cell nuclear transfer (SCNT). Strong RFP expression was detected in the blastocysts and genital ridges of SCNT fetuses but not in other tissues. Two viable transgenic piglets were also produced by SCNT. Reprogramming of fibroblasts from the fetuses and piglets by another round of SCNT resulted in tdTomato reactivation in reconstructed blastocysts. Result indicated that a KI porcine reporter system to monitor the pluripotent status of cells was successfully developed.

## Introduction

Genetically modified pigs have wide applications in both agriculture and biomedicine. However, germline-competent porcine pluripotent stem cells (PSCs) have not been generated yet, which impedes the genetic modification of modified pig models. One potential solution to address this issue is to generate a fluorescence reporter for porcine PSCs.

The transcription factor Oct4, also named *POU5f1*, maintains cell pluripotency [1], thus, the expression of which reflects stem cells pluripotency [2]. Oct4 reporter systems in pigs have been reported previously [3, 4]. However, knowledge on the genomic structure of porcine Oct4 promoter is limited; hence, mouse Oct4 promoter was used to control EGFP expression [3], and 4000 bp upstream of porcine Oct4 start codon was also used as Oct4 promoter [4]. The mouse Oct4 promoter may not interact well with the porcine transcriptional regulatory factor, and the 4000 bp constitutive promoter may not contain all the regulatory elements. Mouse or porcine Oct4 promoter-based florescence reporter systems in pigs were established by randomly integrating the exogenous reporter vector into the porcine genome. The incomplete regulatory region of the Oct4 promoter in the constitutive expression vectors and the uncertain integration sites of these vectors may affect its accuracy of regulation function. Additionally, the variation in copy number and risk of endogenous gene disruption may further affect the reporter gene expression. Precise insertion of a reporter gene into the endogenous Oct4 frame, which allows endogenous promoter-induced reporter gene expression without altering endogenous Oct4 expression, may enhance the accuracy of porcine Oct4 expression simulation.

The knock-in (KI) of a foreign gene into the genome of porcine somatic cells had been difficult because of the extremely low-efficiency of traditional homologous recombination (HR) [5]. Recently, a site-specific nuclease clustered regularly interspaced short palindromic repeats (CRISPR)/CRISPR-associated 9 (CRISPR/Cas9) system was engineered to target and edit specific genome sites assisted by a synthetic single-guide RNA (sgRNA) with simple base-pair complementarity[6]. The CRISPR/Cas9 system has been used to edit the genomes of various organisms, including prokaryotes and eukaryotes [7–11]. Sequence-specific nucleases can efficiently create double stranded breaks in the DNA and significantly increase the frequency of HR through homology-directed repair [7, 12, 13].

The present study generated Oct4-2A-tdTomato reporter piglets through the CRISPR/Cas9 system by precisely replacing the stop codon of Oct4 with a 2A-tdTomato fragment. This endogenous Oct4 promoter-based reporter system is expected to improve the accuracy of monitoring pluripotency of pig cells during embryonic development and cell reprogramming, thereby optimizing the protocols for pig cloning and PSC line generation.

## Materials and Methods

### Animals and ethics statement

The Banna mini-pig, a strain of Chinese indigenous pigs [14], served as cell donor. This study was carried out in strict accordance with the guidelines of Institutional Animal Care and Use Committee of Guangzhou Institute of Biomedicine and Health, Chinese Academy of Sciences (Animal Welfare Assurance #A5748-01). The protocol was approved by the Institutional Animal Care and Use Committee of Guangzhou Institute of Biomedicine and Health, Chinese Academy of Sciences (ID 2012040) and the Department of Science and Technology of Guangdong Province (ID SYXK 2005–0063). For surgery, pigs were induced anesthesia using propofol (2 mg/kg), and given further anesthetic under anesthesia machines (O2 flux: 3L/min, the concentration of isoflurane: 3%). For euthanasia, concentrated barbiturate drugs (500 mg/kg) are given through vein injection to cause a progressively deeper state of anesthesia until cardiac arrest occurs. All efforts were made to minimize the suffering.

### Plasmid construction

CMV-Cas9 vector which has selectable markers neomycin was a gift from George Church (#41815, Addgene). U6-sgRNA cloning vector was constructed by introducing two BbsI restriction sites into the plasmid gRNA_GFP-T1 (#41819, Addgene). The sgRNAs were designed to target the locus near the stop codon of porcine Oct4 by GN19NGG rule, which were constructed as previously described [8]. The primer sequences were designed as follows: caccGTGCCTGCCCTTCCCAGGAG and aaacCTCCTGGGAAGGGCAGGCAC. U6-gRNA was amplified by the primers gctcaattgACCAAGGTCGGGCAGGAAG and gctactagtAGAAAAAAAGCACCGACTCGG, and cloned into the CMV-Cas9 vector by using MfeI and SpeI, and formed final vector C9-gPO. The homologous arms were amplified by genomic PCR and cloned into the pUC19 vector. Subsequently, P2A sequence was ligated with the tdTomato gene and inserted between the right and left arms. The Sequence of donor was list as follow: HA-R:*2A*:***tdTomato***:HA-L

TTCTAGGGCCACACCCGCAGCAAATGGAGGTTCCCAGGCTAGGGGTCCAATCGGAGCTATAGCTGCCAGCCTACGCCAGAGCCACACACAGCCATGTGGGATCTGAGCCACGT…….…GGGCCCTCACTTCACCACCCTGTACTCCTCGGTCCCATTCCCTGAGGGTGAGGCCTTTCCCTCGGTGTCTGTCACCCCTCTGGGCTCCCCCATGCATTCAAACgtcgac*GGAAGCGGAGCTACTAACTTCAGCCTGCTGAAGCAGGCTGGAGACGTGGAGGAGAACCCTGGACCT*cgaccggtcgccacc***ATGGTGAGCAAGGGCGAGGAGGTCATCAAAGAGTTCATGCGCTTCAAGGTGCGCATGGAGGGCTCCATGAACGGCCACGAGTTCGAGATCGAGGGCGAGGGCGAGGGCCGCCCCTACGAGGGCACCCAGACCGCCAA***………**.*CCCGTGCAACTGCCCGGCTACTACTACGTGGACACCAAGCTGGACATCACCTCCCACAACGAGGACTACACCATCGTGGAACAGTACGAGCGCTCCGAGGGCCGCCACCACCTGTTCCTGTACGGCATGGACGAGCTGTACAAGTAG***gtaccttaagtatctagaAGTGGGGGGGGGTGAGGAAGGGGTGAGCTAGGGAGAGAAGCCTGGGGTTTGTACCAGGGCTTTGGGATTAAGTTCTTCATTCACTAAGAAAGGAATTGGGAACACAAAGGGTGTGGGGGCAGGGAGTCTAGGGGAACTGGTTGGAGGGAAGGTGAAGTTCAATGATGCTCTTGATTTTAATCCCCAC………TTGCATATCACCTCAACCAGAAACTCCATCTTGTGTTTTTTTTTCTTTTGGTTGTCCCTGAAGCACGTGGAAATTCCTGGGCCAGAGACTGAACCCTTGCCACCCCTGTAACCCTTGCCACAGCAGTGACAACACTGGAGCCTTAACTTGCTGAGCCACCAGA

### Isolation and culture of PFFs

Primary PFFs were isolated from 35-day-old fetuses of Chinese Banna mini-pig Inbred line. After removing heads, tails, limbs and viscera, the fetuses were cut into small pieces and digested with collagenase IV-DNase in cell culture medium containing 0.32 mg/mL collagenase IV and 2500 IU/mL DNase for 4–6 h at 39°C. Isolated PFFs were cultured in 10 cm dishes for another 12 h and then frozen in fetal bovine serum (FBS) containing 10% dimethylsulfoxide for further use.

### Transfection and selection of KI positive colonies

One day before transfection, PFFs were thawed and cultured in 6 cm dishes. Approximately 1 × 10^5^ PFFs were suspended in 10 μL suspension Buffer R containing 0.2 μg of C9-gPO and 0.8 μg of donor, these PFFs were transfected at 1350 V, 30 ms and 1 pulse number with the Neon™ transfection system (Invitrogen). The transfected cells were distributed equally into ten 100-mm dishes. After recovery for 24 h, G418 (800 μg/mL) was added in the culture medium. The selection medium was changed every other day for one week. Ten days after electroporation, Cell colonies were harvested and cultured in 48-well plates. When the colonies grew to about 1 × 10^5^ cells, part of each colony was lysed in lysis buffer containing NP-40 (0.45%, Sigma) and proteinase K (1mg/ml, TAKARA) at 56°C for 20 min, followed by 90°C for 5 min. Cell lysates were used as genomic templates to detect the integration of exogenous genes by PCR. Transgenic positive colonies were served as donor cells for SCNT.

### SCNT

The protocols used for porcine oocyte collection, in vitro maturation and SCNT were similar to those employed in our previous studies [10, 15]. Oocytes that matured *in vitro* were collected and enucleated using a beveled glass pipette by aspirating the first polar body and the metaphase II plate in a small amount of surrounding cytoplasm in a manipulation medium of HEPES-buffered M199 plus cytochalasin B (7.5 μg/mL). The donor cells were injected into the perivitelline cytoplasm of enucleated oocytes by using the same slit in the zona pellucida as made during enucleation. Two DC pulses at 1.2 kV/cm for 30 μs using an electrofusion instrument successively fused and activated the produced embryos. Some reconstructed embryos were cultured and allowed to develop in vitro up to the blastocyst stage. The blastocysts was measured under fluorescent microscope (X51, Olympus) and determined by genomic PCR as described above. The reconstructed embryos with highly efficient tdTomato expression were marked. Their original donors were further used for SCNT, and the reconstructed embryos were transferred to the oviducts of surrogates after overnight culture in PZM3 at 39°C. Ultrasonography was adopted to monitor the pregnancy status of the surrogates weekly until delivery.

### *In vitro* porcine blastocysts culture

The reconstructed embryos were cultured in the PZM3 for6–7 days after SCNT. Embryos developed until the expanded blastocysts showed hatched or hatching morphology along with a large blastocoele cavity. Zona pellucidae were removed and transferred into Tyrode’s solution. Repeated pipetting was performed using an ultrafine insulin syringe attached to a 29-Gauge needle. Subsequently, blastocysts were collected and planted on mitomycin C-treated MEF feeder layers in porcine ESC medium consisting of α-MEM supplemented with 10% KnockOut™ Serum Replacement (KSR), 5% FBS, 2 mM GlutaMax, 1% non-essential amino acids, 0.1 mmol/l β-mercaptoethanol, 10 ng/ml recombinant human FGF-basic, 10 ng/ml recombinant human EGF-basic, 10 ng/ml Activin A and 10 ng/ml recombinant human leukemia inhibitory factor (LIF, Millipore).

### Generation of piPS-LCs

piPS-LCs were induced in accordance with a previously described protocol[16]. Briefly, human Oct4, Sox2, Klf4 and c-Myc were separately cloned into lentiviral vector FUGW (Addgene, 14883), and the constructs FUW-hOct4, FUW-hSox2, FUW-hKlf4 and FUW-hc-Myc were packaged into a virus in 293T cell lines by co-transfecting with auxiliary packaging vectors (psPAX2 and pMD2.G). After 2 days, the reconstructed lentivirus were collected and centrifuged in a SW28 swinging bucket rotor (Beckmann, USA) at 80,000 ×g for 2 h at 4°C, supernatant was then carefully removed and the pellets were suspended in Opti-MEM^®^ reduced serum medium at 4°C overnight.

PFFs were seeded at 1 × 10^4^ cells per well in a 12-well plate. On the next day, each concentrated virus was added to the medium with MOI (MOI = viral titer/cell number) ranging from 50 to 80 for each lentivirus and then incubated for 24 h. Three days after the initial viral transduction, the cells were digested and seeded onto MEF feeder layers. On day 4, PFF medium was replaced with porcine ESC medium for further culture. After 7–11 days, piPS colonies were harvested and plated onto new plates for further culture.

### Genomic PCR, RT-PCR, and Q-PCR

**Fig 1:**
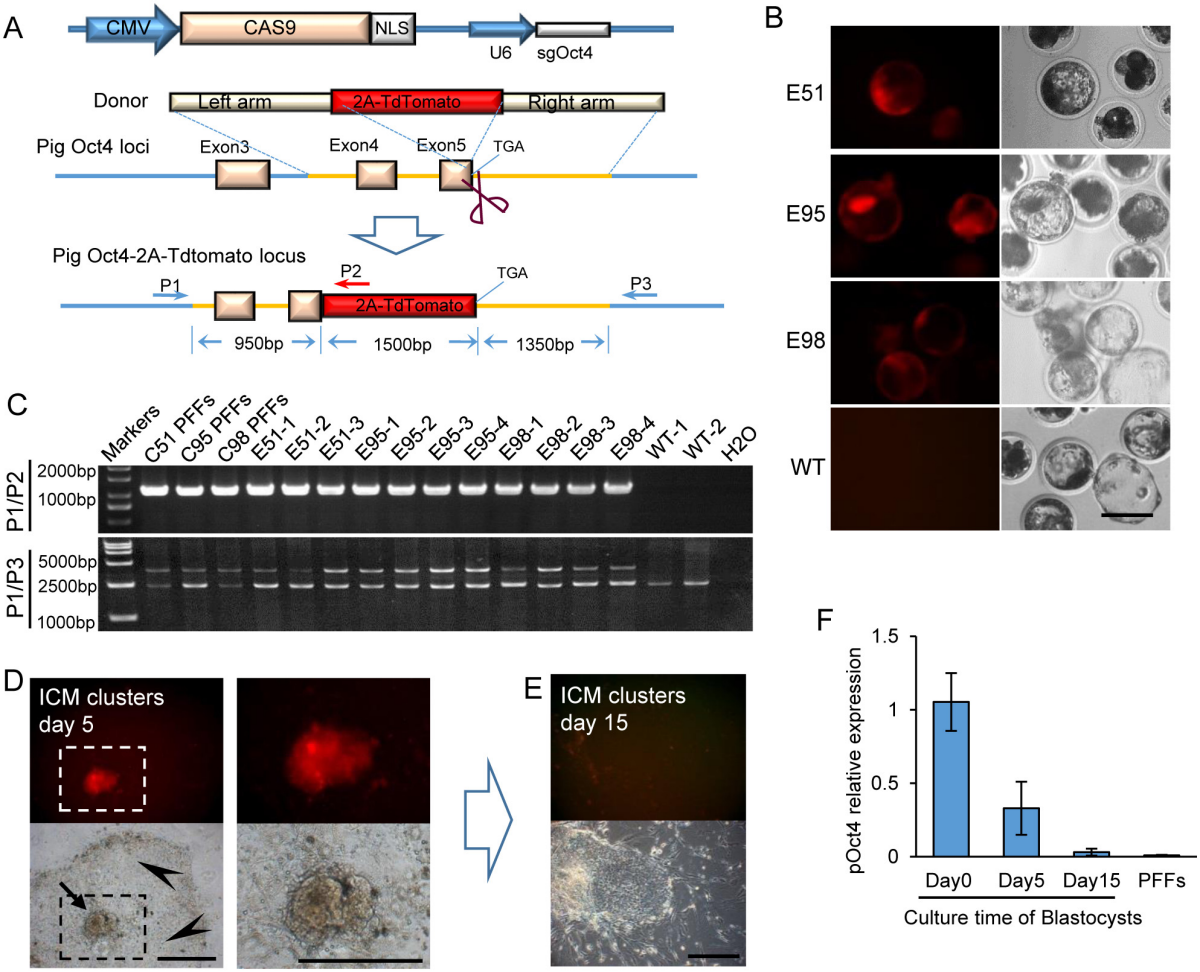
Generation of an endogenous porcine Oct4-2A–tdTomato reporter. (A) Schematic of the method in generating porcine Oct4-2A–tdTomato KI allele. The genes Cas9 and sgOct4 were constructed in a single vector. The homologous arms of the donor vector are indicated as left arm (950 bp) and right arm (1350 bp). PCR primers (P1, P2 and P3) used for PCR genotyping are indicated by blue and red arrowheads. (B) Red fluorescence in porcine Oct4-2A–tdTomato reconstructed blastocysts derived from SCNT embryos. The porcine fibroblast colonies C51, C95, and C98 were reprogrammed to become highly efficient tdTomato-expressing blastocysts. Scale bar, 100 μm. (C) PCR genotyping using the primers indicated in the diagram detected the KI colones and embryos. PCR revealed wild type band (0 bp) and the 2A-tdTomato KI band (1000 bp) using primers P1/P2. PCR revealed the WT band (2300 bp) and the 2A-tdTomato KI band (3900 bp) using primers P1/P3. (D) The targeted porcine blastocysts remained tdTomato positive in the ICM (arrow), whereas disappeared in trophoblast cells (arrowhead), after 5 days cultured in ES medium. Scale bar, 50 μm. (E) The targeted porcine blastocysts differentiated into tdTomato negative colonies after being cultured for 15 days. Scale bar, 50 μm. (F) Q-PCR compared the endogenous Oct4 mRNA levels of porcine gene-target blastocysts cultured for 5 and 15 days. PFFs were used as negative control and porcine blastocysts without being cultured were used as positive control.

Cell lysates from fibroblasts or extracted genome from tissues were used as genomic templates in PCR. To detect the integration of the 2A-tdTomato fragment, the primers were 5ʹ- gctcttccttttggaggcagtgg -3ʹ (P1), 5ʹ—cttcagcctgctgaagcaggc -3ʹ (P2), and 5ʹ—ctaaaacacggggggttagtgggtc -3ʹ (P3). The positions of the primers are shown in Fig 1A. To detect the integration of Cas9 fragment, primers were designed as 5ʹ- GGAGGAGGATAAAAAGCACGAG -3ʹ (C9F), 5ʹ—GATAAGATTACCAAACAGGCCG -3ʹ (C9R).

Porcine blastocysts cultured for 0, 5 and 15 days were collected. Total RNA was extracted from each sample by using Total RNA Kit II (Omiga). First-strand cDNAs were synthesized using a PrimeScript^®^ RT reagent Kit (RR047A, TAKARA) in accordance with the manufacturer’s instructions. RT-PCR reactions were performed using Premix Ex Taq™ Version 2.0 (TAKARA). Primer sequences were: GAPDH (sense: 5’- tggctacagcaacagggtggtg -3’, antisense: 5’- gatggaaactggaagtcaggagatg -3’); endogenous Oct4 (sense: 5’- ggccctcacttcaccaccctg -3’, antisense: 5’- ttctctccctagctcaccccttc -3’); exogenous Oct4 (sense: 5’- ctcacttcactgcactgtac -3’, antisense: 5’- gtattcttaactatgttgctc -3’). Quantitative PCR (Q-PCR) reactions were performed using SYBR^®^ Premix Ex Taq II (RR820A, TAKARA). Primer sequences were: pGAPDH (sense: 5’- aaggtcggagtgaacggatttg -3’, antisense: 5’- gtcttctgggtggcagtgatgg -3’); pOct4 (sense: 5’- gctcactttgggggttctctttg -3’, antisense: 5’- tgcttgatcgtttgcccttctg -3’)

## Results

### Establishment of PFFs with tdTomato reporter in the endogenous Oct4 gene

A Cas9-sgRNA vector targeting the Oct4 gene was constructed to insert a specific reporter gene into the endogenous Oct4 locus. A double-stranded vector to connect P2A-tdTomato reporter with the last codon of the porcine Oct4 gene was used as the donor sequence in HR (Fig 1A). The vectors were cotransfected into primary PFFs from a female fetus. After one-week G418 selection, a total of 223 colonies were picked and seeded onto 48-well plates, and 184 colonies grew to confluence. A small part of cells from each colony were collected for genomic PCR (data not shown). Twenty one colonies (11.41%, 21/184) showed precise integration of the 2A-tdTomato reporter by sequencing (Table 1). Cells that can proliferate well from these colonies served as nuclear donors for somatic cell nuclear transfer (SCNT). The reconstructed blastocyst rate was 10~30%, within the normal range (Table 2), indicating that the in vitro development of nuclear-transferred embryos was not adversely affected by the reporter gene integration.

**Table 1:** Selection of the tdTomato-positive KI positive colonies.

Total colonies	Analyzed colonies	KI positive by PCR (%)	Colonies for SCNT	tdTomato activation (%)
223	184	21 (11.41)	19	8 (42.11)

**Table 2:** Efficiency of generation of tdTomato-positive reconstructed embryos using genotyping KI positive colonies as nuclear donors.

Donor cells	Reconstructed embryos	Blastocysts (%)	tdTomato-positive blastocysts (%)
C7	35	6 (17.14)	3 (50.0)
C39	41	7 (17.07)	2 (28.57)
C51	99	26 (26.26)	17 (65.38)
C95	439	109 (24.83)	95 (87.16)
C98	158	37 (23.42)	28 (75.67)
C123	41	10 (24.39)	4 (40.00)
C149	174	21 (12.07)	6 (28.57)
C161	53	12 (22.64)	1 (8.33)
Wild type	165	39 (23.64)	0

### Validation of tdTomato reporter function in porcine blastocysts

The tdTomato reporter was expressed in NT blastocysts derived from 8 cell lines (8/21, 38.10%) (Table 2 and Fig 1B). About 8–90% of the blastocysts from each cell line expressed tdTomato. The tdTomato-expressing blastocysts derived from C51, C95 and C98 were genotyped by genomic PCR to confirm successful KI at the endogenous loci. To ensure specificity for the endogenous locus, the forward primer (P1) was specified for the genomic sequence beyond the left homology arm, and two reverse primers were set; one (P2) was designed within 2A-tdTomato present in the donor plasmids and the other (P3) was specified for the genomic sequence beyond the right homology arm. The PCR products of the P1/P2 primers, which was expected to be a 1000 bp DNA fragment, can be used to test the specific integration site. The PCR product of P1/P3 primers was used to further investigate whether both alleles were integrated with 2A-tdTomato. A 2300 bp band for the wild-type (WT) genome, a 3900 bp band for the homozygotes, and both 2300 and 3900 bp bands for the heterozygotes were expected (Fig 1A). As shown in Fig 1C, 2A-tdTomato was precisely integrated at the Oct4 gene loci (**upper row**) in one of the alleles (**lower row**) of blastocysts derived from C51, C95, and C98. To identify whether Cas9 vector was integrated into genome, all of three colonies were amplified by genomic PCR. We found that C51 and C95 colonies were integrated with Cas9 sequence, while C98 were free of Cas9 sequence (S1A Fig).

A total of 153 NT blastocysts emitting red fluorescence were cultured in ES medium. The embryos were attached to the bottom of the plates. After 5 days, ICMs from derived some blastocysts formed a dome-like cluster and trophoblast cells spread out around the ICMs. Cells of ICM clusters were fluorescent but trophoblast cells were not (Fig 1D). The glowing ICM clusters were passaged to a new dish seeded with feeder cells for another 10 days. Given the extended culture, the fluorescence in the ICM cells gradually become faint, and cell proliferation ceased (Fig 1E). Q-PCR analysis confirmed that Oct4 was eventually silenced in the ICM derived cells after 15 days (Fig 1F). The ICM derived cells from all 153 NT blastocysts failed to undergo two passages.

### Analysis of cloned fetuses

**Fig 2:**
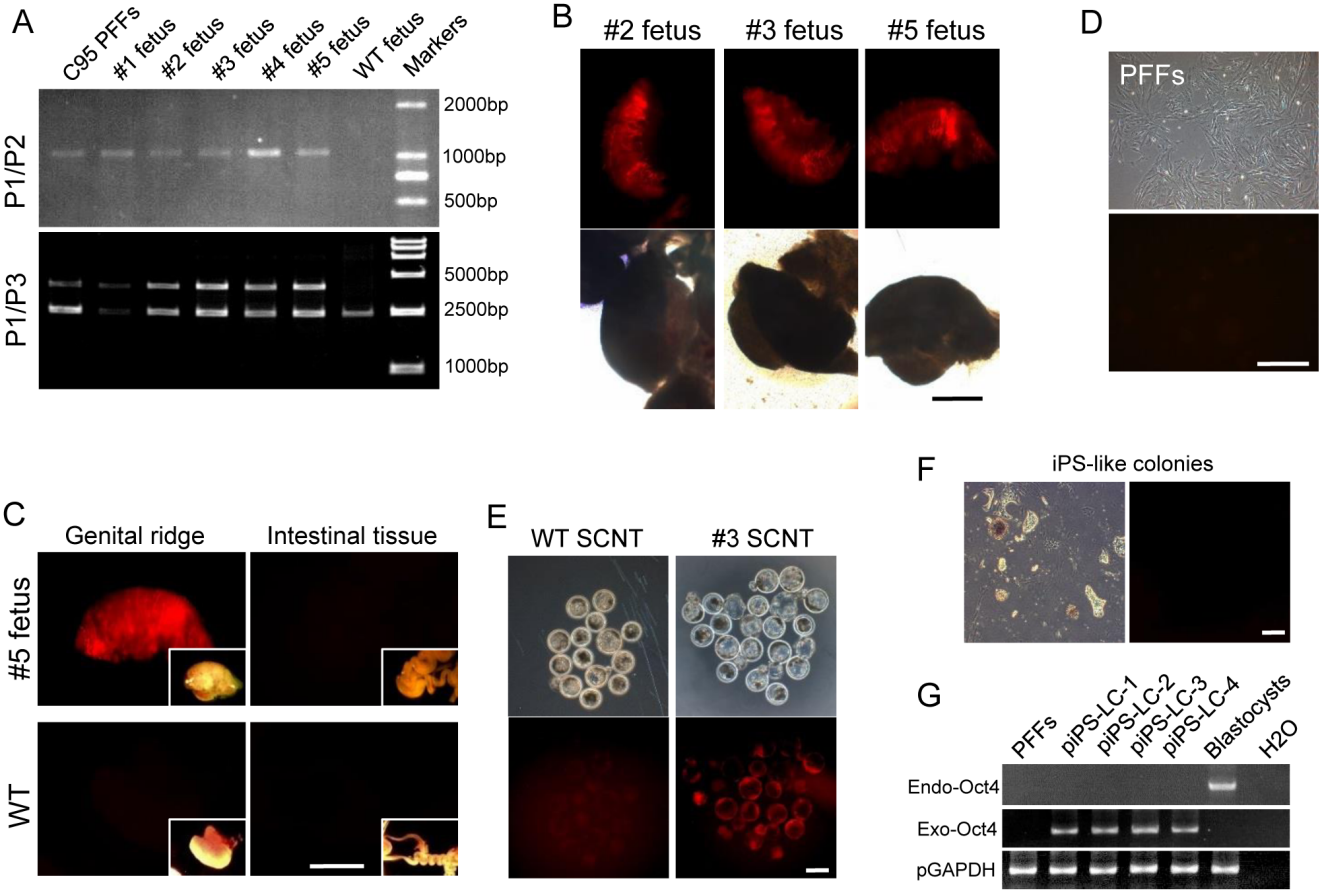
Analysis of the KI fetuses. (A) PCR genotyping detected the KI fetuses by using primers indicated in the diagram. PCR revealed wild type band (0 bp) and the 2A-tdTomato KI band (1000 bp) using primers P1/P2. PCR revealed the WT band (2300 bp) and the 2A-tdTomato KI band (3900 bp) using primers P1/P3. (B) TdTomato was expressed, with varying degree of fluorescence, in PGCs of genital ridges isolated from fetuses. (C) TdTomato was expressed in the genital ridges of target fetuses, but not in other tissues, such as intestines. tdTomato was not expressed in WT genital ridges. Bright field image of the tissue is shown on the lower right corner of each image. Scale bar, 1 mm. (D) PFFs cultured in vitro did not express tdTomato. Scale bar, 100 μm. (E) tdTomato was expressed in blastocysts after another round of SCNT using fibroblasts isolated from pOct4-2A-tdtoamto KI fetuses as nuclear donors. WT blastocysts did not express tdTomato. Scale bar, 100 μm. (F) Dome-like colonies formed 10 days after transfection did not express tdTomato. Scale bar, 50 μm. (G) RT-PCR was used to identify the transcription of endogenous and exogenous Oct4 in piPS-LCs. All colonies expressed exogenous Oct4 but not endogenous Oct4.

The colonies C51, C95, and C98 showed the highest rate of fluorescent blastocysts, and thus were chosen to conduct SCNT for embryo transfer to generate cloned fetuses and piglets. At 35 days after embryo transfer, one of the pregnancy surrogates transferred with C95 embryos was terminated, and a total of five fetuses (denoted as #1 to #5) were retrieved by cesarean section. Genomic PCR with P1/P2 primers showed 1000 bp band in all fetuses (Fig 2A, **upper row**), indicating that 2A-tdTomato was precisely integrated at the Oct4 gene loci. Genomic PCR with P1/P3 primers revealed both 2300 and 3900 bp bands in all five fetuses (Fig 2A, **lower row**), indicating that these fetuses were heterozygotes. Genital ridges were collected and observed under a microscope. The tdTomato expression was found in the genital ridges of these fetuses (Fig 2B). By contrast, tdTomato was not expressed in other tissues, such as the intestines (Fig 2C).

### Reprogramming of somatic cells

The KI porcine PFFs were isolated from the five fetuses respectively (Fig 2D), and used as nuclear donors for another round of nuclear transfer. The tdTomato protein was expressed in both ICM and trophoblasts of all the blastocysts (Fig 2E), indicating that the cells from all the five fetuses contained the functional Oct4 reporter system.

The PFFs were infected with lentiviral vectors encoding four human transcription factors (Oct4, Sox2, Klf4, and c-Myc), the essential transcription factors for iPSC induction. The iPS-like colonies with multi-layer compacted cells were formed 10 days after infection. These iPS-like cells [named porcine induced PS-like cells (piPS-LCs)] can undergo long-term passages. However, no tdTomato expression was observed in any initial iPS-like colonies and long-term cultured piPS-LC lines of up to 16 passages (Fig 2E). Reverse transcription PCR (RT-PCR) analysis showed that exogenous Oct4, instead of endogenous Oct4 was expressed in the piPS-LCs lines (Fig 2F).

### Production and analysis of OT piglets

**Fig 3:**
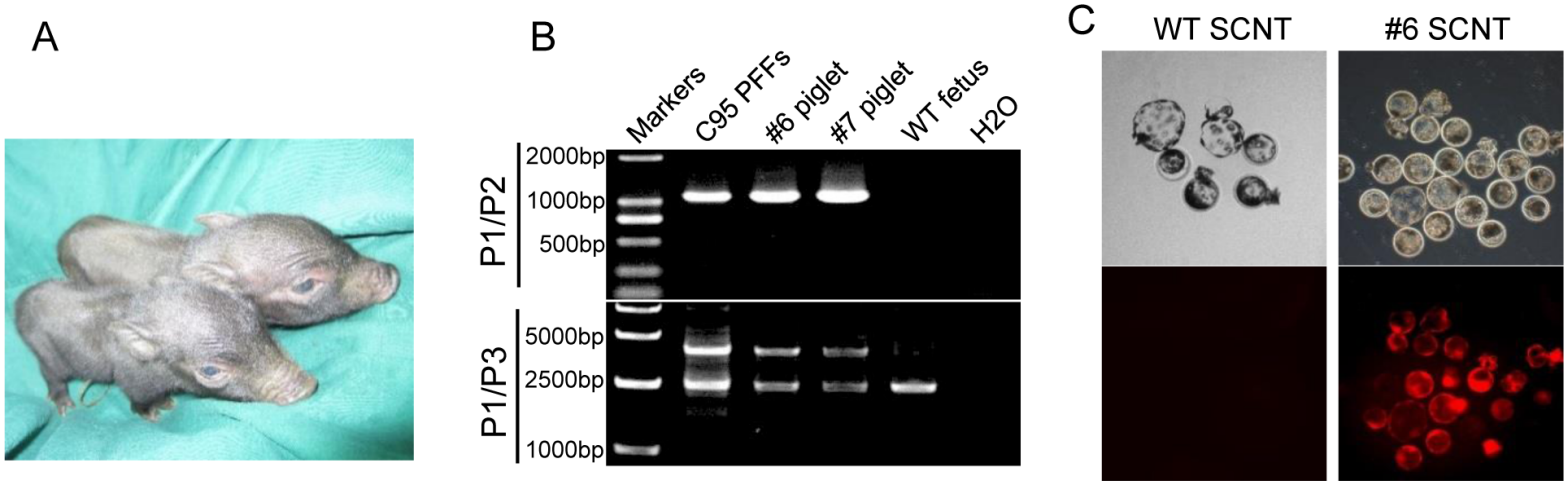
Analysis of the KI piglets. (A) Two cloned piglets were derived from reconstructed embryos using C95 cells as nuclear donors. (B) PCR genotyping using the primers indicated in the diagram detected KI in piglets. PCR revealed wild type band (0 bp) and the 2A-tdTomato KI band (1000 bp) using primers P1/P2. PCR revealed the WT band (2300 bp) and the 2A-tdTomato KI band (3900 bp) using primers P1/P3. (C) tdTomato expression was reactivated in blastocysts after the second SCNT using fibroblasts isolated from pOct4-2A-tdtoamto KI piglets as nuclear donors. WT blastocysts did not express tdTomato. Scale bar, 100 μm.

The reconstructed embryos developed to full term in one surrogate. Two cloned piglets (denoted as #6 and #7) were delivered by natural birth 113 days after embryo transplantation (Table 3 and Fig 3A). PCR was used to detect the 2A-tdTomato sequence from the genomic DNA extracted from the ear punch tissues of new born piglets. As shown in Fig 3B, P1/P2 primers revealed a 1000 bp band, and P1/P3 primers revealed 2300 and 3900 bp bands, indicating that both piglets were heterozygotes. Fibroblasts isolated from the ear punch tissues, were used as nuclear donors to perform a second round of nuclear transfer. The tdTomato protein was expressed in both ICM and trophoblasts of all the blastocysts (Fig 3C).

**Table 3:** Summary of nuclear transfer results using tdTomato-positive KI positive fibroblasts.

Donor cells	Reconstructed embryos	Embryos transferred	Recipients	Pregnant	Live piglets
C51	103	103	1	1	0
C95	246	246	2	2^a^	2
C98	253	253	2	1	0

Note:

^a^one of the 2 pregnancy surrogates was terminated after 35 days embryo transfer.

### Integration of Cas9 vector into porcine genome

Genome from fetuses and piglets were collected for PCR. PCR primers were designed to amplify exogenous Cas9. We found that Cas9 sequences were integrated into the genome of all fetuses and piglets cloned from C95 cells (S1B Fig). Furthermore, we found that the fibroblasts from all cloned fetuses and piglets were G418 resistant (S1C Fig), indicating that the C9-gPO vector were integrated into the genome of OT KI fetuses and piglets.

## Discussion

The establishment of porcine ES cells and iPSCs is not at par with that in mice. One of the reasons is attributed to the limited knowledge on the mechanisms underlying the differentiation and reprogramming of pig embryos and cells. A pig model with the Oct4 reporter system, which monitors differentiation and reprogramming states similar to those in murine iPSCs and ESCs, is beneficial to investigate the differentiation and reprogramming of pig embryos and cells. In this study, CRISPR/Cas9 technology was employed to successfully generate pigs carrying the tdTomato reporter in endogenous porcine Oct4 gene loci via HR combined with SCNT. The fluorescent fluorescence reporter is driven by the endogenous Oct4 promoter, thus, fluorescence accurately reveals the endogenous Oct4 activation.

The P2A sequence connected the Oct4 and tdTomato genes, allowing the cotranslational “cleavage” of their proteins [17]. Thus, once the endogenous Oct4 promoter is reactivated, the Oct4 and tdTomato protein would be translated independently and would not functionally interfere with each other in the same cell.

HR is typically used in gene function analysis of mouse embryonic stem cells. However, no ES cell lines from large animals, including pigs, have passed the crucial test of germline contribution. SCNT combined with HR remains to be the sole method for gene KI in these species. This method is highly inefficient, and only few attempts succeed because of the limited proliferation competency and extremely low frequency of HR in somatic cells (<10^−6^) [5, 18]. The current study employed the CRISPR/Cas9 system to efficiently insert exogenous DNA sequences through HR. PCR results showed that 21 out of 223 colonies exhibited precise integration of the reporter gene. Given that no positive (e.g., antibiotic resistance) and/or negative selection markers (e.g., diphtheria-toxin) existed in the homologous vector, an acceptable 10% efficiency far exceeds the previous HR in somatic cells [5, 18].

SCNT is an effective approach to reprogram a differentiated cell into a pluripotent one. Therefore, SCNT is a rapid and simple system to verify the functionality of our KI Oct4-RFP reporting system. Red fluorescence is observed once somatic cells with Oct4-RFP KI were reprogrammed into NT embryos containing PSCs. Cells of the 21 positive colonies served as donor cells, and tdTomato was expressed in NT blastocysts derived from eight cell lines, although the rate of tdTomato-expressing blastocysts was variable, that probably because not all cells in a colony may have originated from a single fibroblast, and some non-targeting cells sneaked into the colonies.

After G418 selected for 7 days, two of the three colonies were integrated with exogenous Cas9 gene. But further study on SCNT, we found the cloned fetuses and piglets did not demonstrate any adverse consequences, proving that the integration of Cas9 does not affect the survival of animal [19].

One of the most important applications of the Oct4 reporter system is to monitor status of the pluripotency of isolated ESCs and somatic cells induced into pluripotent cells. Attempts were made to isolate ES cell lines by using up to 153 glowing reconstructed blastocysts derived from the fibroblasts with the Oct4-RFP reporter system. The fluorescence of ICM cells gradually became faint, indicating loss of pluripotency. When the PFFs were infected with lentiviral vectors encoding four human transcription factors, iPS-like cells can be cultured for an extended period. However, tdTomato expression was not expressed in any initial iPS-like colonies or cell lines cultured for an extended period. RT-PCR assay also confirmed that endogenous Oct4 was not activated, indicating that exogenous Oct4, rather than endogenous Oct4, initiated and maintained the pluripotency of iPS-like cell lines. Similar to the outcomes of previous efforts to establish porcine iPS cells [20–22], the current results indicated that the culture condition to induce and maintain porcine pluripotent cells has not been optimized yet.

In summary, CRISPR/Cas9-mediated genome engineering was adopted to successfully generate KI pigs carrying Oct4-tdTomato reporter. This reporter system can precisely reflect porcine Oct4 expression in real-time. This is the first time to establish a reporter system in large animal by KI technique, which provides a simple and practical way for studying the mechanism of early embryonic development, the differentiation of germline stem cells, and the derivation and maintenance of pluripotent cells in pigs.

## Supporting Information

S1 Fig Integration of CRISPR/Cas9 expression vector into porcine genome. (A) Genome PCR for integration of Cas9 sequence in selected clones C51, C95 and C98. Markers: DL2000; Cas9 PFFs: the porcine fetal fibroblasts transient transfected with CRISPR/Cas9 expression vector; WT PFFs: wild type PFFs. (B) Genome PCR for integration of Cas9 sequence in fetuses and piglets. Markers: DL2000; Cas9 PFFs: the porcine fetal fibroblasts transient transfected with CRISPR/Cas9 expression vector; WT PFFs: wild type PFFs. (C) Fetal and piglet fibroblasts from C95 clones cultured in medium containing G418 (800 μg/mL) for 7 days. All fetal and piglet fibroblasts from C95 clones grew well in G418 medium. Wild type PFFs, as control, died all after cultured in G418 medium for 7 days.
(PDF)

## References

[1] Shi G, Jin Y. Role of Oct4 in maintaining and regaining stem cell pluripotency. Stem Cell Res Ther. 2010;1(5):39 10.1186/scrt39 21156086 PMC3025441

[2] Froschauer A, Khatun MM, Sprott D, Franz A, Rieger C, Pfennig F, et al oct4-EGFP reporter gene expression marks the stem cells in embryonic development and in adult gonads of transgenic medaka. Mol Reprod Dev. 2013;80(1):48–58. 10.1002/mrd.22135 . 23139203

[3] Nowak-Imialek M, Kues WA, Petersen B, Lucas-Hahn A, Herrmann D, Haridoss S, et al Oct4-enhanced green fluorescent protein transgenic pigs: a new large animal model for reprogramming studies. Stem Cells Dev. 2011;20(9):1563–75. 10.1089/scd.2010.0399 . 21126163

[4] Huang L, Fan N, Cai J, Yang D, Zhao B, Ouyang Z, et al Establishment of a porcine Oct-4 promoter-driven EGFP reporter system for monitoring pluripotency of porcine stem cells. Cellular reprogramming. 2011;13(2):93–8. 10.1089/cell.2010.0069 . 21254851

[5] Lai L, Kolber-Simonds D, Park KW, Cheong HT, Greenstein JL, Im GS, et al Production of alpha-1,3-galactosyltransferase knockout pigs by nuclear transfer cloning. Science. 2002;295(5557):1089–92. 10.1126/science.1068228 . 11778012

[6] Jinek M, Chylinski K, Fonfara I, Hauer M, Doudna JA, Charpentier E. A programmable dual-RNA-guided DNA endonuclease in adaptive bacterial immunity. Science. 2012;337(6096):816–21. 10.1126/science.1225829 . 22745249 PMC6286148

[7] Cong L, Ran FA, Cox D, Lin S, Barretto R, Habib N, et al Multiplex genome engineering using CRISPR/Cas systems. Science. 2013;339(6121):819–23. 10.1126/science.1231143 23287718 PMC3795411

[8] Mali P, Yang L, Esvelt KM, Aach J, Guell M, DiCarlo JE, et al RNA-guided human genome engineering via Cas9. Science. 2013;339(6121):823–6. 10.1126/science.1232033 23287722 PMC3712628

[9] Bikard D, Euler CW, Jiang W, Nussenzweig PM, Goldberg GW, Duportet X, et al Exploiting CRISPR-Cas nucleases to produce sequence-specific antimicrobials. Nature biotechnology. 2014;32(11):1146–50. 10.1038/nbt.3043 . 25282355 PMC4317352

[10] Zhou X, Xin J, Fan N, Zou Q, Huang J, Ouyang Z, et al Generation of CRISPR/Cas9-mediated gene-targeted pigs via somatic cell nuclear transfer. Cell Mol Life Sci. 2014 10.1007/s00018-014-1744-7 . 25274063 PMC11113635

[11] Niu Y, Shen B, Cui Y, Chen Y, Wang J, Wang L, et al Generation of gene-modified cynomolgus monkey via Cas9/RNA-mediated gene targeting in one-cell embryos. Cell. 2014;156(4):836–43. 10.1016/j.cell.2014.01.027 . 24486104

[12] Yang H, Wang H, Shivalila CS, Cheng AW, Shi L, Jaenisch R. One-step generation of mice carrying reporter and conditional alleles by CRISPR/Cas-mediated genome engineering. Cell. 2013;154(6):1370–9. 10.1016/j.cell.2013.08.022 23992847 PMC3961003

[13] Krentz NA, Nian C, Lynn FC. TALEN/CRISPR-Mediated eGFP Knock-In Add-On at the OCT4 Locus Does Not Impact Differentiation of Human Embryonic Stem Cells towards Endoderm. PLoS One. 2014;9(12):e114275 10.1371/journal.pone.0114275 25474420 PMC4256397

[14] Zeng R, Zeng YZ. Molecular cloning and characterization of SLA-DR genes in the 133-family of the Banna mini-pig inbred line. Anim Genet. 2005;36(3):267–9. 10.1111/j.1365-2052.2005.01277.x . 15932417

[15] Fan N, Chen J, Shang Z, Dou H, Ji G, Zou Q, et al Piglets cloned from induced pluripotent stem cells. Cell research. 2013;23(1):162–6. 10.1038/cr.2012.176 23247628 PMC3541650

[16] Quan L, Chen Y, Song J, Yan Q, Zhang Q, Lai S, et al Establishment of a Rabbit Oct4 Promoter-Based EGFP Reporter System. PloS one. 2014;9(10):e109728 10.1371/journal.pone.0109728 . 25360692 PMC4215976

[17] Szymczak AL, Workman CJ, Wang Y, Vignali KM, Dilioglou S, Vanin EF, et al Correction of multi-gene deficiency in vivo using a single 'self-cleaving' 2A peptide-based retroviral vector. Nature biotechnology. 2004;22(5):589–94. 10.1038/nbt957 . 15064769

[18] Rogers CS, Stoltz DA, Meyerholz DK, Ostedgaard LS, Rokhlina T, Taft PJ, et al Disruption of the CFTR gene produces a model of cystic fibrosis in newborn pigs. Science. 2008;321(5897):1837–41. 10.1126/science.1163600 18818360 PMC2570747

[19] Platt RJ, Chen S, Zhou Y, Yim MJ, Swiech L, Kempton HR, et al CRISPR-Cas9 Knockin Mice for Genome Editing and Cancer Modeling. Cell. 2014;159(2):440–55. 10.1016/j.cell.2014.09.014 . 25263330 PMC4265475

[20] Ezashi T, Telugu BP, Alexenko AP, Sachdev S, Sinha S, Roberts RM. Derivation of induced pluripotent stem cells from pig somatic cells. Proc Natl Acad Sci U S A. 2009;106(27):10993–8. 10.1073/pnas.0905284106 19541600 PMC2698893

[21] Wu Z, Chen J, Ren J, Bao L, Liao J, Cui C, et al Generation of pig induced pluripotent stem cells with a drug-inducible system. J Mol Cell Biol. 2009;1(1):46–54. 10.1093/jmcb/mjp003 . 19502222

[22] Sui D, Sun Z, Xu C, Wu Y, Capecchi MR, Wu S, et al Fine-tuning of iPSC derivation by an inducible reprogramming system at the protein level. Stem cell reports. 2014;2(5):721–33. 10.1016/j.stemcr.2014.03.013 24936457 PMC4050490

