# Supplementary material for: Generation of Knock-In Pigs Carrying Oct4-tdTomato Reporter through CRISPR/Cas9-Mediated Genome Engineering
Source: PLoS One. 2016 Jan 12;11(1):e0146562. doi: 10.1371/journal.pone.0146562 (PMC4710570; doi:10.1371/journal.pone.0146562)
Supplement: S1 Fig — (A) Genome PCR for integration of Cas9 sequence in selected clones C51, C95 and C98. Markers: DL2000; Cas9 PFFs: the porcine fetal fibroblasts transient transfected with CRISPR/Cas9 expression vector; WT PFFs: wild type PFFs. (B) Genome PCR for integration of Cas9 sequence in fetuses and piglets. Markers: DL2000; Cas9 PFFs: the porcine fetal fibroblasts transient transfected with CRISPR/Cas9 expression vector; WT PFFs: wild type PFFs. (C) Fetal and piglet fibroblasts from C95 clones cultured in medium containing G418 (800 μg/mL) for 7 days. All fetal and piglet fibroblasts from C95 clones grew well in G418 medium. Wild type PFFs, as control, died all after cultured in G418 medium for 7 days. (PDF) [file pone.0146562.s001.pdf]

Supplemental Figure

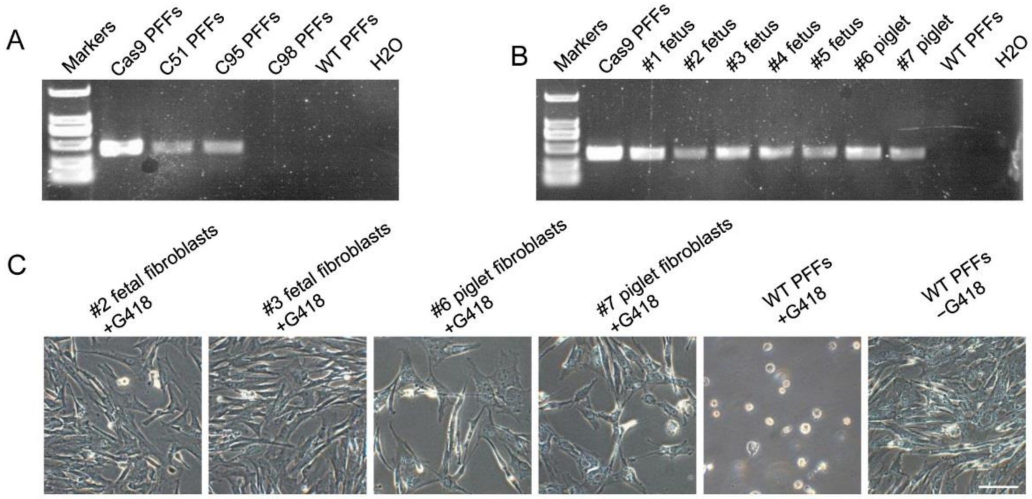

Figure S1. Integration of CRISPR/Cas9 expression vector into porcine genome.

(A) Genome PCR for integration of Cas9 sequence in selected clones C51, C95 and C98. Markers: DL2000; Cas9 PFFs: the porcine fetal fibroblasts transient transfected with CRISPR/Cas9 expression vector; WT PFFs: wild type PFFs.

(B) Genome PCR for integration of Cas9 sequence in fetuses and piglets. Markers: DL2000; Cas9 PFFs: the porcine fetal fibroblasts transient transfected with CRISPR/Cas9 expression vector; WT PFFs: wild type PFFs.

(C) Fetal and piglet fibroblasts from C95 clones cultured in medium containing G418 (800 μg/mL) for 7 days. All fetal and piglet fibroblasts from C95 clones grew well in G418 medium. Wild type PFFs, as control, died all after cultured in G418 medium for 7 days.
